# The impact of obesity on the male reproductive axis

**Published:** 2014-06-25

**Authors:** R Mihalca, S Fica

**Affiliations:** * “Carol Davila" University of Medicine and Pharmacy, Bucharest, Romania; ** Endocrinology and Diabetes Department, “Elias" University Hospital, Bucharest, Romania

**Keywords:** obesity, male hypogonadism, androgen levels, testosterone, weight loss

## Abstract

Abstract

Obesity, defined as a body mass index (BMI) >30 kg/m2, has seen an important increase in prevalence in the last decades, not only in Europe and the United States, but also in developing countries. It is an established risk factor for numerous pathologic conditions like diabetes mellitus, cardiovascular diseases and cancer, but has also been linked to male hypogonadism. Several studies showed a negative impact of excessive BMI on testosterone levels, sexual function and sperm parameters. Possible mechanisms beyond this phenomenon are reduced hypothalamic and pituitary secretory function, excess estrogen production and reduced circulating sex-hormone binding globulin (SHBG). Peptides produced by the adipocyte may also trigger modifications of the reproductive function. Independently of the method used, non-surgical approach or bariatric techniques, weight reduction and a return to a normal BMI have been associated with improvement in the sexual function and levels of sexual hormones in obese males, showing that obesity related hypogonadism is preventable. Sexual and reproductive health might represent additional motivational factors for men in order to maintain a healthy life-style.

## Introduction

In this new century, obesity and overweight represent a major matter of public health. According to the World Health Organization, the worldwide prevalence of obesity has almost doubled since 1980 [**1**]. In 2008, about 35% of the adults above the age of twenty were overweight and 11% were obese [**1**]. Initially considered a condition typically found in high-income societies, obesity turned epidemic also among the developing countries. As a result, more than half of the world's population lives in countries where overweight and obesity have a higher impact on mortality than underweight [**1**]. The growing prevalence of obesity may be attributed to increased intake of high-caloric food and decreased physical activity, common features of the modern lifestyle. Obesity is therefore the result of an imbalance between caloric intake and energy expenditure.

 Obesity has a clear impact on the onset or progression of various diseases like type-2 diabetes, hypertension, osteoarthritis and even cancer [**2**]. Although the consequences on human reproduction are still under discussion, studies on adult males report an association between obesity and impairment of reproductive hormones or sperm parameters [**3**]. The aim of this study is to explore the impact of obesity on the male reproductive system, to review the current mechanisms proposed, and to discuss the prevention strategies. 

 Definition of obesity 

 The most sensitive methods of quantifying human adipose tissue are radiologic: dual energy X-ray absorptiometry, computed tomography or nuclear magnetic resonance. Less precise methods, but easier to use in clinical practice, are anthropometric measures like body mass index (BMI), waist circumference and waist-hip ratio. For men, a waist circumference >102 cm or a waist–hip ratio >0.9 indicate increased body fat and are considered predictors of the occurrence of comorbidities [**4**]. Calculating BMI, the ratio between the body weight in kilograms and the height in meters squared, remains the main method to identify obese patients in both men and woman. The World Health Organization considers overweight a BMI >25 kg/m2 and obesity a BMI >30 kg/m2 [**1**]. 

 Overview of the male reproductive system

 The reproductive function starts with the signal provided by the hypothalamic peptide Kisspeptin [**5**]. It triggers the release of Gonadotropin-Releasing Hormone (GnRH) pulses from the hypothalamus that further stimulates the secretion of gonadotropins (Follicle-Stimulating Hormone [FSH] and Luteinizing Hormone [LH]) by the anterior pituitary. FSH and LH act at the testicular level to induce sperm maturation at the tubular compartment and steroid hormone production at the interstitial compartment. More than 95% of the male androgen hormones derive from the testis and testosterone is the main product of the Leydig cell, with a daily normal output of 6-7 mg [**6**]. Testosterone has a pivotal role in spermatogenesis and in fertile men testicular concentrations are >80-fold higher than blood concentrations. The serum total testosterone is of 44% bound to Sex Hormone Binding Globulin (SHBG) and 54% to Albumin, only about 2% representing the free ratio [**6**]. Testosterone has a key role in triggering male sexual characteristics such as the development of genital organs, muscle and bone mass, and body hair growth. Part of the androgenic effects reflects a direct action of testosterone on target cells, whereas others are the consequence of the peripheral conversion to 5α-dihydrotestosterone and estradiol. 

 Impact of obesity on the male reproductive axis: clinical evidence

 Male hypogonadism is a syndrome that results from the inability of the gonads to provide physiological levels of testosterone and a normal number of spermatozoa (androgen deficiency and male infertility) due to an impairment at one or more levels of the hypothalamic-pituitary-testicular axis [**7**]. Whereas male infertility often triggers the lack of conception, symptoms and signs of androgen deficiency are variable because they can be modified according to age, duration of deficiency, concomitant diseases, replacement therapy or individual perception. Some clinical features of hypogonadism are highly specific (reduced libido, erectile dysfunction, loss of body hair, breast tenderness, hot flushes and sweats), whereas others are less (decreased energy, depressive mood, sleep disturbance, increased body fat and BMI) [**7**].

 While several studies suggest a link between increasing BMI or waist/hip ratio and impairment of seminal parameters, a similar group of studies does not [**3**]. Among 390 men from infertile couples but without a known male infertility factor, Hammoud et al. [**8**] found an odds ratio (OR) for oligozoospermia of 3,3 (95% confidence interval [CI] 1,19-9,14) for obese males compared to patients with normal BMI. Furthermore, the OR of having a progressively motile sperm count <10x106 was 3,4 (95% CI 1,12-10,6). Hofny et al. [**9**] made a comparison between 42 fertile obese and 80 infertile obese men and found a significant difference in BMI between the two groups (mean [kg/m2] ± standard deviation [SD]: 31,21 ± 0,39 versus 33,49 ± 0,43), BMI having a significant negative correlation with sperm count and sperm motility. Stewart et al. evaluated a cohort of 225 Australian fertile men and showed that men with BMI >30 kg/m2 had a significantly lower total sperm count than those with BMI <30 kg/m2 [**10**]. A recent meta-analysis that included 14 studies corresponding to 9779 male subjects concluded that obese men had a higher risk of oligozoospermia (OR 1.42; 95% CI 1.12-1.79) or azoospermia (OR 1.81; 95% CI 1.23-2.66) compared to normal-weight men [**11**]. On the contrary, the cross-sectional population-based study involving 1989 men conducted by Aggerholm et al. did not show any difference in sperm concentration and total sperm count between the obese group and the normal weight group (68 x 106 /ml [95% CI 58-78]) versus 59 x106 /ml [95% CI 55-63], and 190 x106 /ml [95% CI 161-223] versus 168 x106 /ml [95% CI 157-180]) [**12**]. In line with the above results, the meta-analysis conducted by MacDonald et al. concluded that evidences do not show a clear relationship between BMI category and the mean or median sperm concentration, mean or median total sperm count, mean semen volume and average sperm motility [**13**].

 A significant body of evidence makes the relationship between obesity and impairment of reproductive hormones or presence of symptoms of hypogonadism less debated [**3,14**]. Most studies report a reduction in plasma SHBG and total testosterone levels, whereas a weaker relationship is observed between BMI and free testosterone. Estradiol levels can be elevated in obese patients, but MacDonald et al. found a positive relationship with BMI only in four out of the ten studies measuring estradiol [**13**]. As reviewed by Teerds et al., in 88% of the analyzed studies, the decrease in testosterone was not parallel to a reduction in LH and FSH levels and obese males often showed normal gonadotropin levels [**3**]. Erectile dysfunction and reduced libido are also common causes of sexual dysfunction among obese males. Despite the differences in the geographical area of setting and heterogeneity of evaluation methods, a number of eight population based studies showed a higher prevalence of erectile dysfunction in obese males compared to normal-weight subjects [**15**]. To bring an example from the European population, the survey “Contexte de la Sexualité en France" that included 4635 men, found a higher occurrence of erectile dysfunction in obese males (OR 2.58, 95% CI 1.09 - 6.11, p < 0.05) [**16**]. 

 Possible mechanisms beyond the negative effect of obesity on the male reproductive axis 

 A normal energy balance consists of sufficient caloric input, adequate energetic expenditure and efficient thermoregulatory costs. Each level of the reproductive axis (hypothalamus, pituitary gland and gonads) is able to respond to the energetic modifications. In cases of long-term impairment of the energetic balance, the pulsatile secretion of GnRH is modified and subsequent long term effects on the male reproductive function can occur [**17**]. The exact mechanisms underlying the influence of obesity on the male reproductive axis in humans are still under debate.

 As previously stated, total testosterone is often reduced in obese males. Presumably, this imbalance is, at least in part, explained by the reduction in blood SHBG levels, frequently found in obese subjects [**18**]. The negative correlation between BMI and SHBG might be the result of hyperinsulinism, because insulin has shown to be an important inhibitor of hepatic SHBG output [**19**]. Furthermore, plasma SHBG levels return to normal when weight loss is achieved [**20**]. An important issue debated today is whether testosterone and SHBG reduction are clinically evident. If the testicular testosterone output is severely reduced and not parallel to the reduction of SHBG, free testosterone levels could probably also show a decrease [**3**]. 

 Excess adipose tissue might be the cause of increased aromatization of testosterone, resulting in higher estradiol levels [**21,22**]. An increased testosterone/estradiol ratio has been found to be linked to male infertility [**23**]. Visceral adiposity could therefore act as an endocrine-disruptor, reducing gonadotropin secretion through a negative feedback of estradiol at a hypothalamic and pituitary level, establishing a condition of hypogonadotropic hypogonadism [**24**]. This phenomenon probably occurs only in a subgroup of patients, as supported by the rich number of studies that showed normal gonadotropin levels in obese males [**3**].

 A number of at least 30 peptides are produced by the white adipose tissue, most of them with biological effects on the human organism. Some of them are related to the reproductive function in animal models, most likely because of an action at the hypothalamic level [**14**]. Leptin is a 16 kDa polypeptide produced mainly at the adipocyte level but also at the hypothalamic, pituitary, gastric and gonadal level, with important functions in the energetic balance of the organism [**25**]. Leptin acts as a satiety signal and levels increase parallel to weight gain, being a good parameter of body adipose stores [**26**]. An effect on GnRH and LH pulsatile secretion is presumed, as demonstrated by the association with puberty onset [**27**]. In human males, leptin was found negatively correlated to total and free testosterone, also after adjusting for the effect of SHBG, LH and estradiol, resulting a good predictor of low androgen levels in obese subjects [**28**]. Adiponectin is a polypeptide produced exclusively at the adipose level, that reduces hepatic glucose production and increases hepatic insulin sensitivity [14,29]. Obese subjects often show low adiponectin levels, negatively correlated to insulin resistance and risk of type-2 diabetes [**30**]. Adiponectin is thought to exert a permissive effect on LH release because its receptors were found at the hypothalamic and pituitary level [**31**]. 

 Obesity is associated to mechanical factors that have been linked to impairment of the male reproductive function. Excess suprapubic adipose tissue has been initially considered a cause of increased scrotal temperature and a negative effect on spermatogenesis could be the possible consequence [**32**]. Studies that are more recent have advanced the hypothesis that oxidative stress and lipid peroxidation could be the link between genital adiposity and infertility in obese males [33,34]. Sleep apnea, a condition characterized by frequent proximal airway tract obstruction resulting in apneas, hypopneas, oxygen desaturation, and arousals from sleep, is frequently associated with the obesity hypoventilation syndrome [**35**]. A reduction in LH and testosterone, as well as an increase in erectile dysfunction prevalence has been found in obese males that present sleep apnea [36-38].

 Consequences of weight loss 

 The above presented evidences show that obesity has an impact on the male reproductive axis and could trigger infertility or androgen deficiency. Most authors agree that the first step in approaching this condition should be the weight reduction, demonstrating that obesity related hypogonadismais preventable. Only few drugs are approved today in the United States for the treatment of obesity (orlistat, lorcaserin and phentermine topiramate), but none of them has been used yet in male patients with low androgen levels or symptoms of hypogonadism [**39**]. Therapeutic interventions used in surveys that evaluated male androgen status included surgery and lifestyle changes (low caloric diet and physical activity).

 After nine weeks of a very low-calorie diet, the 58 obese men evaluated by Niskanen et al. showed a significant increase in SHBG, total and free testosterone, a trend that has also been maintained after a twelve months of follow-up [**40**]. A randomized single-blind trial that delivered a twenty-four months detailed training on caloric intake and physical activity to 55 obese men with erectile dysfunction showed a higher degree of weight loss and a significant improvement in erectile function scores compared to the control group that received only general information about healthy food choices and physical activity [**41**]. Bariatric surgery, a treatment indicated for morbid obesity (BMI>40 kg/m2 or >35kg/m2 with comorbidities), has shown to provide consistent weight loss and reduction in comorbidities [**42**]. A recent meta-analysis that evaluated the consequences of weight loss strategies on androgenic environment showed that all the surgical techniques have been followed by an increase in the levels of testosterone (mean total testosterone increase 8.73 nmol/L [CI 6.51-10.95]), SHBG and gonadotropins [**43**].

## Conclusions

Obesity is a complex medical condition associated with chronic pathological conditions that have an increased risk of mortality. Important modifications in the quality of life are also commonly encountered in obese subjects, and part of them could be related to the sexual and reproductive dysfunction. A significant group of evidences presented to date showed that obese males are more likely to develop androgen deficiency and reduced fertility. On the other hand, cohorts of obese subjects do not report symptoms of hypogonadism and have fathered their own offspring. A possible explanation of this disagreement is that a higher testicular output of testosterone, as seen in young adult males, could maintain a normal androgenic activity. Furthermore, female fertility could compensate the impairment in sperm parameters, providing the achievement of a pregnancy. Therefore, data available today seem to argue that obesity represents a risk factor for male sexual and reproductive dysfunctions. Improvement in reproductive hormones are expected after reducing body fat mass and this finding should be a further motivational factor to induce obese men to lose weight. 
